# Identification of an RNase that preferentially cleaves A/G nucleotides

**DOI:** 10.1038/srep45207

**Published:** 2017-03-21

**Authors:** Jumin Xie, Zhen Chen, Xueyan Zhang, Honghe Chen, Wuxiang Guan

**Affiliations:** 1 Center for Emerging Infectious Diseases, Wuhan Institute of Virology, Chinese Academy of Sciences, Wuhan 430071, Hubei, China; 2 University of Chinese Academy of Sciences, Beijing, 100049, China

## Abstract

Ribonucleases play an important role in the RNA metabolism which is critical for the localization, stability and function of mature RNA transcripts. More and more ribonucleases were discovered in recent years with the progress of technology. In the present study, we found that the uncharacterized C19orf43, a novel interacting protein of human telomerase RNA (hTR), digested T7 transcribed RNA, total cellular RNA and RNA oligos but not DNA. Thus we named this new RNase as hTRIR (human telomerase RNA interacting RNase). Genetic analysis showed that hTRIR is conserved among eukaryotic species and widely expressed in different cell lines. The RNase activity of hTRIR works in a broad temperature and pH range while divalent cations are not required. The conserved C-terminus of C19orf43 is necessary for its activity. Finally, we found that hTRIR cleaves all four unpaired RNA nucleotides from 5′ end or 3′ end with higher efficiency for purine bases, which suggested that hTRIR is an exoribonuclease. Taken together, our study showed the first evidence of the novel function of hTRIR *in vitro*, which provides clue to study the regulatory mechanism of hTR homeostasis *in vivo*.

The metabolism of RNA transcripts requires a large number of distinct ribonucleases which includes endoribonucleases and exoribonucleases^1^,^2^,^3^. Following identification and characterization of the bovine pancreatic RNase A^4^,^5^, more and more ribonucleases and homologs were discovered in different organisms with the explosion of genomic sequences in recent years^6^,^7^,^8^,^9^. In addition to the RNA cleavage, ribonucleases were reported to have other function such as cancer-suppress^10^, anti-tumor effect^11^,^12^,^13^, antiviral activities^14^ and can be acted as antimicrobial reagents^15^,^16^.

The uncharacterized human protein C19orf43 is located at Chromosome 19: p12 and encodes a protein of 176 amino acids with a molecular weight of 18,419 Dalton^17^,^18^. C19orf43 mRNA is widely expressed in human organs such as brain, spinal cord and bone marrow, etc (http://www.genecards.org/cgi-bin/carddisp.pl?gene=C19orf43). C19orf43 is highly expressed in nasal epithelium, peripheral blood mononuclear cells, breast, and T lymphocyte. An acetylation site was detected at lysine-146 in human C19orf43 protein with large scale analysis in cellular proteomics by mass spectrum, but the function of this modification is unknown^19^. C19orf43 protein was associated with RNA prespliceosomal A complexes^20^ and pulled down by ZBTB1 which is a transcriptional repressor and promotes chromatin remodeling^21^. However, the function of C19orf43 is unclear yet.

SNRPA, RPL36, UbC and SF3A2 have been reported to interact with C19orf43^22^. SNRPA was a small ribonucleoprotein and a component of the spliceosome^23^. SF3A2 was a splicing factor which may anchor U2 snRNP to the pre-mRNA^24^,^25^. *UbC* is a stress-inducible polyubiquitin gene and maintains cellular ubiquitin (Ub) levels during stress^26^.

Human telomerase RNA (hTR) is one of the subunits of telomerase holoenzyme^27^. hTR provides a scaffold for the assembly of the telomerase and contains a template region that is reverse transcribed by the catalytic subunit which adds telomeric repeats to chromosome ends^28^,^29^. The 3′ half of hTR harbors a hairpin-hinge-hairpin-ACA (H/ACA) motif which is also found in a class of snoRNAs^30^,^31^,^32^. Despite overall similarity with snoRNAs, hTR biogenesis has been suggested to involve a specific yet poorly defined pathway^33^,^34^.

To identify the 3′ end processing proteins of hTR, HeLa-S3 cell nuclear extract (NE) was prepared and partially purified by heparin, cation exchange and gel filtration chromatography. The uncharacterized C19orf43 protein was found in the crude purified complex by mass spectrum. Genetic analysis showed that C19orf43 is conservative among eukaryotic species. Incubation of C19orf43 with T7 transcribed RNA resulted in the degradation of RNA, which suggested that C19orf43 possessed ribonuclease activity. Thus we named this protein as hTRIR (human telomerase RNA interacting RNase). Further study showed that hTRIR not only cleaved T7 transcribed RNA but also total cellular RNA. The RNase activity of hTRIR worked in a broad pH range from 6.5 to 12 and divalent cation was not required. The C-terminus contains the key domain for RNase digestion activity. Importantly, hTRIR cleaves all four RNA nucleotides from 5′ or 3′ end with preference for A/G, suggesting that hTRIR is a novel exoribonuclease.

## Results

### hTRIR was detected in partial purified hTR 3′ end processing complex

**Figure 1:**
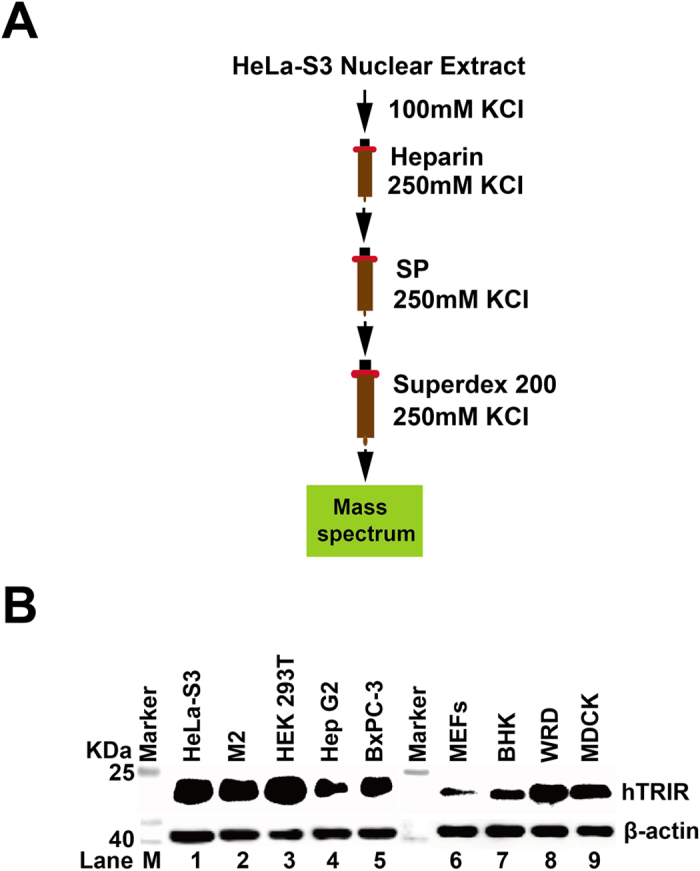
hTRIR was found in the partially purified nuclear extract. (**A**) Purification of nuclear extract. Nuclear extract was purified by heparin column, cation exchange column and gel filtration column as diagrammed. (**B**) Western blot. Cells were harvested and probed with hTRIR polyclonal antibody.

Proper 3′ end processing of a RNA nascent transcript by endoribonuclease or exoribonuclease is critical for the localization, function and stability of the mature RNA^35^,^36^. To investigate what endoribonuclease or exoribonuclease is involved in the 3′ end processing of the non-coding hTR, nuclear extract (NE) was prepared from large scale cultured 50 L HeLa-S3 cells. Purification of hTR 3′ end processing complex was performed as diagrammed (Fig. 1A). NE was first purified by Heparin column to obtain nucleic acid binding proteins, followed by cation exchange column and gel filtration chromatography to get the crude hTR processing proteins. The protein composition of the purified NE was determined by mass spectrum.

The purified hTR processing complex contains 488 proteins. Most of the proteins are splicing factors, nucleases, and helicases, etc. Interestingly, the uncharacterized protein hTRIR was found in the complex. hTRIR was detected in most of human organs and tissues (http://www.proteinatlas.org/ENSG00000123144-C19orf43/tissue). To further confirm the expression of hTRIR protein, polyclonal antibody against hTRIR was developed by immunization of rabbit with purified protein. Several cell lines were cultured and total protein was harvested to detect hTRIR expression by using western blot (Fig. 1B). hTRIR was detected in mammalian cell lines such as HeLa-S3, M2, HEK293T, HepG2, BxPC-3, MEFs, BHK, WRD and MDCK cells, which indicated that hTRIR was widely expressed in eukaryotic cells.

### Genetic analysis of hTRIR

**Figure 2:**
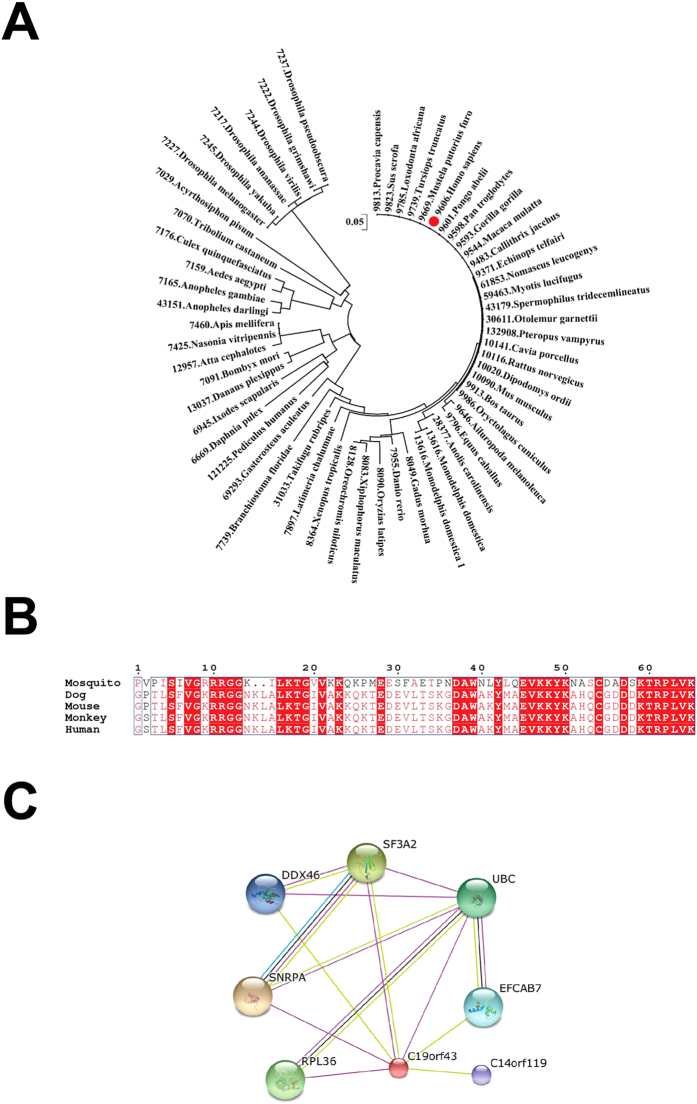
Genetic analysis of hTRIR. (**A**) The phylogenetic tree of hTRIR was constructed by Mega 6.0. (**B**) Sequence alignment of hTRIR C-terminus by clustal W. (**C**) Interacting proteins of hTRIR predicted by STRING.

To investigate the potential function of hTRIR, we searched the orthologous proteins of hTRIR in the database EggNOG^37^. Total 63 orthologous proteins were found to be distributed in 61 eukaryotic species, but no ortholog was found in bacteria or archaea. The sequences of hTRIR from different species were aligned and phylogenetic tree was constructed by using MEGA 6.0 Evolutionary Genetics software^38^, which showed that hTRIR sequence was highly conserved (Fig. 2A). We performed amino acid sequence analysis of hTRIR by Clustal W^39^ and found that the N terminal of hTRIR is heterologous while the C-terminal is highly conserved (Fig. 2B).

We next did text mining by STRING^22^ (http://www.string-db.org/) (Fig. 2C) to search other proteins interacting with hTRIR in addition to the reported SNRPA, RPL36, UbC and SF3A2. DDX46, EFCAB7 and C14orf119 were predicted to interact with hTRIR. DDX46 played an essential role in RNA splicing and rRNA biogenesis^40^. The function of EFCAB7 and C14orf119 were unknown. Four of seven proteins interacting with hTRIR are involved in the biogenesis of RNA, which implicated that the function of hTRIR could be linked to RNA metabolism.

### hTRIR digested different forms of RNA but not DNA

**Figure 3:**
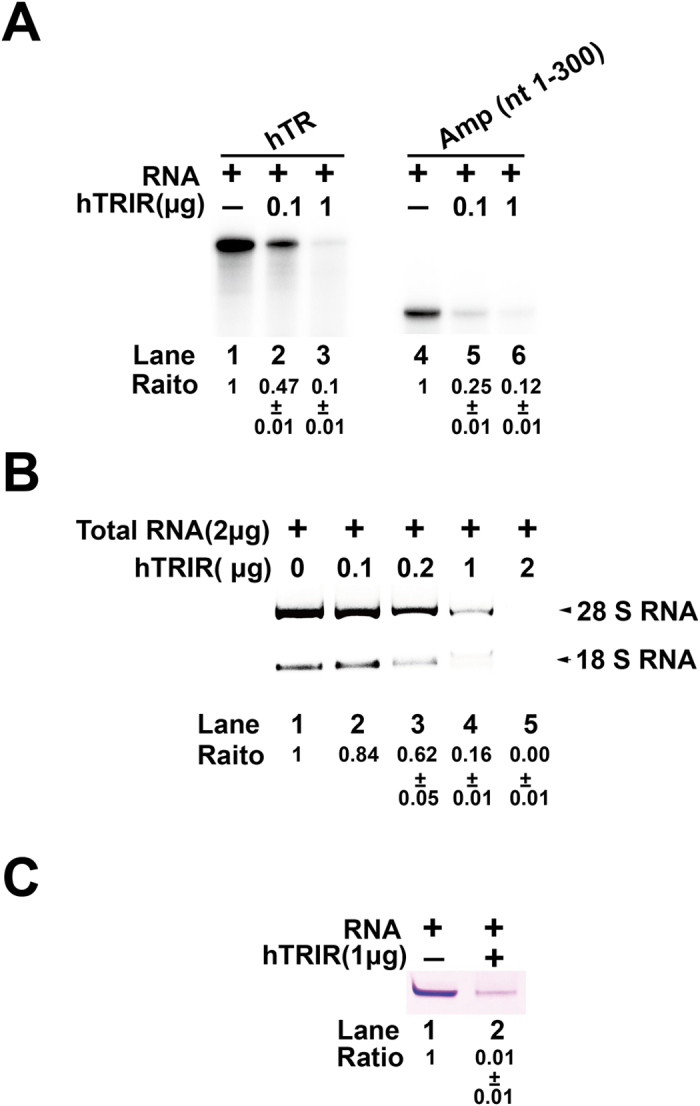
hTRIR digests different forms of RNA by *In vitro* digestion assay. (**A**) T7 transcribed α-^32^P-GTP labeled hTR or ampicillin resistance gene ORF (nt 1–300) was incubated with different amount of purified hTRIR in digestion buffer at 37 °C for 30 min. RNA was purified by phenol-chloroform extraction and precipitated with ethanol-glycogen, then separated by 7 M urea 6% Polyacrylamide gel electrophoresis (PAGE) at 550 V, 15 W for 90 min. Signals were detected with Typhoon Cyclone. (**B**) 2 μg total RNA was incubated with different amount of purified hTRIR at 37 °C for 30 min and separated by 1.5% agarose gel. Signals were detected with GelDoXR + IMAGELAB. (**C**) Small RNA was incubated with purified 1 μg hTRIR at 37 °C for 30 min and separated by 7 M urea 20% PAGE at 200 V, 2 W for 30 min. RNA was stained with stain-all (Sigma) and scanned with Epson scanner.

To test the hypothesis that hTRIR is involved in the RNA metabolism, hTR was transcribed by T7 polymerase *in vitro* and incubated with 0.1 μg or 1 μg purified hTRIR at 37 °C for 30 min in the digestion buffer (NaCl 25 mM, 2% glycerol, 0.5 mM Tris-HCl, pH 7.5). RNA was separated by 7 M urea 6% polyacrylamide gel electrophoresis (PAGE) and the signal was detected by Typhoon cyclone. To our surprise, 53% of T7 transcribed hTR was degraded when 0.1 μg hTRIR was incubated with hTR and over 90% of the transcripts were digested by 1 μg hTRIR in the absence of ATP (Fig. 3A). hTR is a noncoding RNA which contains secondary structure at the 3′ end^41^. To check whether non-structured RNA transcripts can be digested by hTRIR, the first 300 nt of ampicillin resistance gene open reading frame, which does not contain hairpin structure, was transcribed by T7 polymerase and incubated with hTRIR. The result showed that transcribed ampicillin resistance gene RNA was degraded by hTRIR (Fig. 3A).

*In vitro* T7 transcribed RNA does not contain 5′ cap and poly (A) tail. Whether hTRIR digests cellular RNA is unknown. To this end, 2 μg total RNA from HeLa-S3 cells were incubated with 0.1, 0.2, 1 and 2 μg hTRIR. Over 99% 28 S and 18 S RNAs were degraded when the amount of hTRIR increased to 2 μg (Fig. 3B). Incubation of chemically synthesized small RNA (nt = 30) with hTRIR resulted in 99% RNA degradation (Fig. 3C).

**Figure 4:**
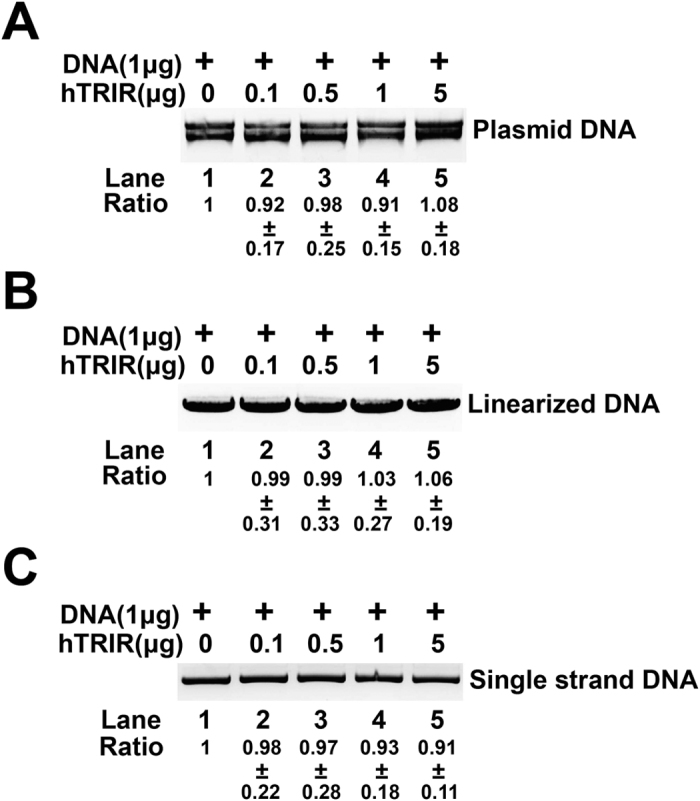
hTRIR does not digest DNA. (**A**) to (**C**) *In vitro* DNA digestion with purified hTRIR. Supercoiled DNA (**A**), linearized DNA (**B**) or single-stranded DNA (**C**) were incubated with purified hTRIR and then separated by 1% agarose gel.

We next checked whether hTRIR could digest DNA. To this end, supercoiled plasmid, linearized plasmid, and single-stranded DNA were incubated with different amount of hTRIR (Fig. 4). The signal of DNA had little change even the amount of hTRIR increased to 5 μg (Fig. 4), which indicated that hTRIR could not digest DNA *in vitro*.

Collectively, our result showed that hTRIR is a novel RNase which digests T7 transcribed RNA, rRNA, RNA oligos but not DNA.

### Optimization of RNA digestion by purified hTRIR

**Figure 5:**
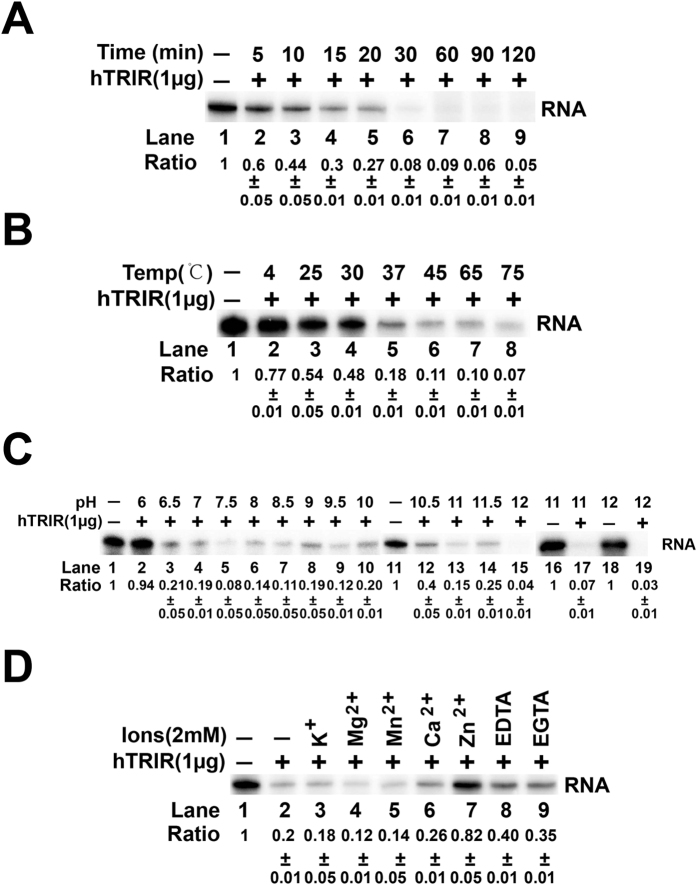
*In vitro* optimization of hTRIR RNA digestion assay. *In vitro* digestion assay was optimized by different incubation time (**A**), temperature (**B**), pH (**C**), ions (**D**). Experiments were repeated at least three times, the quantification data was presented by mean and standard deviation.

To optimize the *in vitro* RNA digestion of hTRIR, T7 transcribed RNA was incubated with hTRIR under different conditions to check the digestion efficiency. First, we incubated 1 μg hTRIR with RNA in digestion buffer for different time. 56% of RNA was digested after 10 min and 92% of RNA was digested after 30 min (Fig. 5A), which demonstrated that the RNA digestion of hTRIR is efficient. Therefore, RNA and hTRIR were incubated for 30 min in the following experiments. We then tested the effect of different temperatures on RNA digestion efficiency. 23% of RNA was degraded under 4 °C and 50% of RNA was still detectable at 25 °C and 30 °C (Fig. 5B), which suggested that the digestion efficiency of hTRIR was not high under low temperature. However, when the temperature reached 37 °C or above, over 80% of RNA was degraded (Fig. 5B). To exclude the possibility of RNA instability under high temperature, we incubated the RNA in the digestion buffer under 65 °C and 75 °C (Fig. 5B). The result showed that RNA was stable under 65 °C and 75 °C (Fig. 5B).

To test the effect of pH on the digestion efficiency of hTRIR, pH of digestion buffer was adjusted from 6 to 12. Most of RNA was digested by hTRIR when pH was between 6.5 and 12 and RNA showed stability under pH 11 to 12 (Fig. 5C). Our results showed that the RNase activity of hTRIR worked in a broad pH range.

The concentration of different ions is important for the activity of RNase. Therefore, K^+^, Mg^2+^, Mn^2+^, Ca^2+^, Zn^2+^ and chelating agents EDTA, EGTA were added to the digestion buffer to test the effect on the activity of hTRIR (Fig. 5D). Zn^2+^ suppressed hTRIR RNA digestion ability (Fig. 5D). K^+^, Mg^2+^, Mn^2+^, Ca^2+^, EDTA and EGTA had no influence on hTRIR RNA digestion efficiency (Fig. 5D).

### The key digestion domain of hTRIR

**Figure 6:**
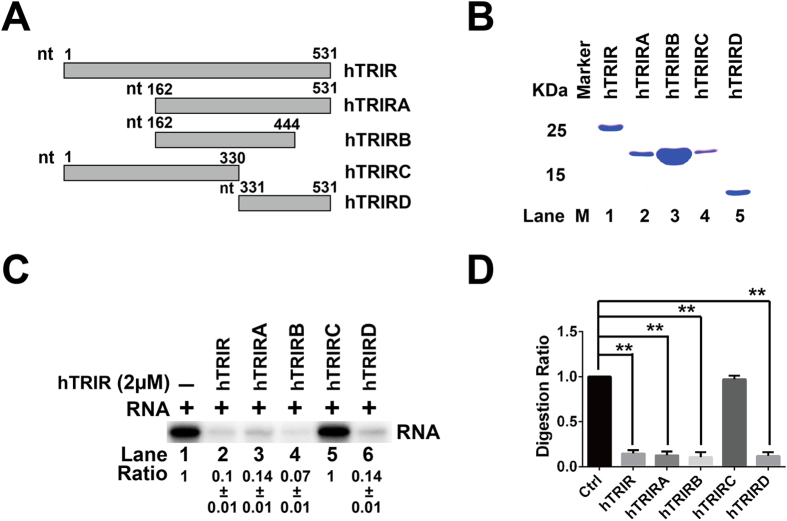
The C terminus of hTRIR contains key RNase domain. (**A**) Truncated plasmids of hTRIR as diagrammed. (**B**) Purified truncated proteins. Plasmids diagrammed in A were transformed to BL21 and the expressed protein was purified. Proteins were separated by 15% SDS-PAGE and stained with Commassie blue. (**C**) RNase digestion assay. Truncated hTRIR proteins were incubated with T7 transcribed RNA and signals were detected by Typhoon Cyclone. (**D**) Statistical analysis of digestion efficiency of different hTRIR mutants in (**C**). Experiments were repeated at least three times and student *t* test were performed. Values of p < 0.05 were considered as statistically significant and marked with *.

Sequence analysis showed that N terminal of hTRIR has low homology while the C terminal harbors a conserved region (Fig. 2B). To investigate the potential functional domain of hTRIR, hTRIRA to hTRIRD were constructed by insertion of different part of hTRIR into pET-33b(+) as diagrammed (Fig. 6A). Truncated proteins were purified (Fig. 6B) and RNA digestion assay were performed in the digestion buffer at 37 °C for 30 min. The result showed that hTRIRA, hTRIRB and hTRIRD possessed the RNA digestion activity while the hTRIRC did not (Fig. 6C and D). As shown in Fig. 6A, hTRIRC does not contain the C terminus while hTRIRA, hTRIRB and hTRIRD contain the C terminus. These data suggested that the highly conserved C terminus is crucial to the RNA digestion function of hTRIR.

### hTRIR digests purine bases in the RNA

**Figure 7:**
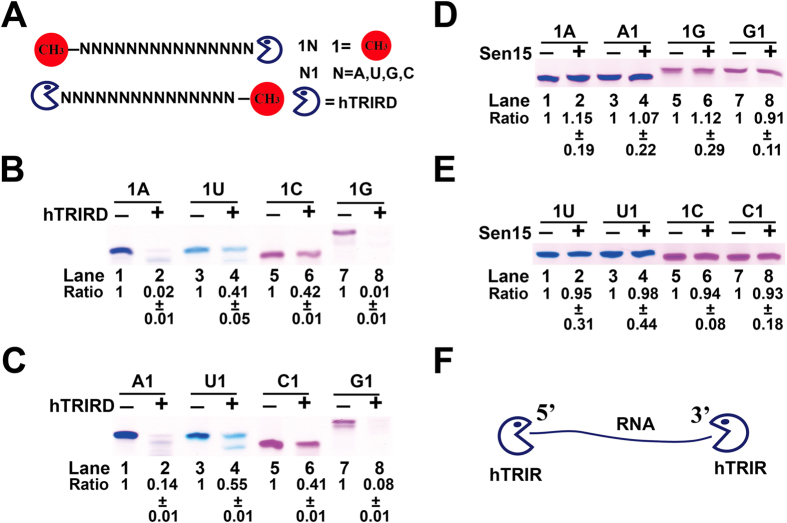
hTRIR cleaves RNA from 5′ end or 3′ end. (**A**) Chemically synthesized RNA oligos with methylated group at either 5′ end or 3′ end were shown. (**B**) and (**C**) *In vitro* RNA digestion assay. RNA oligos with methylated group at 5′ end or 3′ end were incubated with hTRIR and separated by 7 M urea 20% PAGE. RNA was stained with stain-all and scanned with Epson scanner. (**D**) and (**E**) *In vitro* RNA digestion assay. RNA oligos with methylated group at 5′ end or 3′ end were incubated with Sen15 and separated by 7 M urea 20% PAGE. RNA was stained with stain-all and scanned with Epson scanner. (**F**) The cartoon of potential mechanism of hTRIR in RNA digestion.

To investigate what bases are digested by hTRIR and how hTRIR cleaves RNA, RNA oligos with terminal methylated at 5′ end or 3′ end were synthesized (Fig. 7A) and incubated with hTRIRD which has the similar function of hTRIR to check the digestion efficiency (Fig. 6C and D). Incubation of hTRIRD with oligo(A) or oligo(G) with methylated group at either 5′ end or 3′ end resulted in degradation of the oligos (Fig. 7B and C). Approximate 50% of oligo(U) and oligo(C) were digested by hTRIRD (Fig. 7B and C). Those results indicated that hTRIRD is an exoribonuclease which cleaves all four nucleotides in the RNA. To exclude the possibility of RNA degradation and RNase contamination, we incubated the synthesized RNA oligos with purified Sen15 which had the same purification process as hTRIR. No RNA cleavage was observed, which suggested that no RNase contaminated during our purification process. Those data support that hTRIR is an exoribonuclease.

## Discussion

In the present study, the uncharacterized protein hTRIR was identified in the crude 3′ end processing complexes of hTR. hTRIR is ubiquitously expressed and conservative among eukaryotic species. Further study showed that hTRIR digested T7 transcribed RNA and cellular total RNA *in vitro* but not DNA. The RNase activity of hTRIR works well at temperature between 37 °C and 75 °C, broad pH range from 6.5 to 12. Interestingly, Zn^2+^ inhibited the RNase activity of hTRIR while Mg^2+^, Ca^2+^, Mn^2+^ K^+^, Na^+^, EDTA and EGTA had little effect on the exoribonuclease activity of hTRIR. The C terminus of hTRIR contains key domain which is responsible for the RNA digestion activity. Finally, we demonstrated that hTRIR harbors exoribonuclease activity which cleaves purine bases from 5′ end or 3′ end with higher efficiency than pyrimidines.

hTRIR protein was detected in the partially purified 3′ end processing complexes of hTR by mass spectrum. Phylogenetic analysis and sequence alignment showed that hTRIR is conserved among eukaryotic species and the C terminus contains a conservative region which may play an important role in the function of hTRIR. The truncated proteins hTRIRA, hTRIRB and hTRIRD, which contain the C terminus, have the same exoribonuclease activity as hTRIR. However, hTRIRC, which does not contain the C terminal region, could not digest RNA. These results suggested that the C terminal region is responsible for RNA digestion activity.

Most proteins interacted with hTRIR are involved in the biogenesis of RNA, which indicated the function of hTRIR is linked to RNA metabolism^23^,^24^,^25^. To elucidate the function of hTRIR, purified hTRIR was incubated with T7 transcribed hTR, total cellular RNA, short RNA oligos and DNA. To our surprise, RNAs were cleaved by hTRIR but DNA was not, which suggested that hTRIR has the function of exoribonuclease but no function of DNase.

The digestion condition was optimized by different time, pH and ions since hTRIR worked in the digestion buffer which contains 25 mM Na^+^, 2% glycerol and 0.5 mM Tris-HCl under pH 7.5. Interestingly, Zn^2+^ inhibited the digestion activity. Mg^2+^, which is included in most of digestion buffer, is not required for the activity. K^+^, Ca^2+^, Mn^2+^, EDTA and EGTA had no influence on hTRIR RNA digestion ability. To exclude the possibility of RNase contamination during hTRIR purification, we incubated RNA with Sen15, TOE1 and FEN1 which have the similar purification process as hTRIR (TOE1 and FEN1 cleavage assay data not shown). No RNA cleavage was observed when RNA was incubated with those proteins, which indicated that it was the function of hTRIR resulting in the degradation of RNA.

Different RNases cleave RNA with different mechanism^42^,^43^. In our study, hTRIR cleaved A and G residues efficiently from 5′ or 3′ end, indicating that it contains exoribonuclease activity. Unpaired pyrimidine residues were cleaved by hTRIR with lower efficiency than purine bases. The characteristic of hTRIR is different from the known RNase A, T1, T2, U2 and H. RNase A cleaves unpaired pyrimidine residues on the single-stranded RNA^5^,^44^, and RNase T1 cleaves 3′ end unpaired G residue^45^. RNase T2 cleaves all RNA residues but prefers A residue^46^. RNase U2 specifically cleaves unpaired A residue^47^. RNase H cleaves DNA/RNA duplex to produce ssDNA via hydrolytic mechanism^48^,^49^.

RNase A super-family members not only cleaves RNA, but also suppresses cancer and tumor^10^,^11^,^12^ and restrains microorganism growth^50^. RNase H had been developed as drug target for blocking virus replication^51^,^52^. Our results demonstrated that hTRIR immunoprecipitated with hTR and digested T7 transcribed hTR *in vitro*. However, whether hTRIR played a role in the biogenesis of hTR *in vivo* is still unknown. Further study showed that hTRIR was able to cleave cellular total RNA and RNA oliogs *in vitro*. Whether hTRIR is involved in other cellular process is unclear yet. Our study does not reveal the key amino acid for hTRIR, which is still under investigation.

In conclusion, hTRIR protein is conservative among eukaryotic species and possesses the function of exoribonuclease *in vitro*. How hTRIR affects the cell and whether it participates in other cellular process need to be elucidated in the future. Our study provided the clue to look into the function of hTRIR *in vivo*.

## Methods

### Ethics statement

This study was performed in strict accordance with recommendations in the Guide for the Care and Use of Laboratory Animals according to the regulation in the People’s Republic of China. Based on “Guideline for Animal Care and Use, Wuhan Institute of Virology (WIV), Chinese Academy of Sciences (CAS)”, The animal used in the experiment was bred and maintained under specific pathogen-free conditions at the Central Animal Laboratory of Wuhan Institute of Virology, Chinese Academy of Sciences (WIV, CAS, Lincese number: SYXK2014–0034). All animal experiments were approved by the Institutional Animal Ethical Committee of WIV, CAS (Approval Number: WIVA32201401). All experimental protocols were approved by the Ethical Committee of WIV, CAS.

### Cell lines and cell culture

HeLa-S3 (ATCC, CCL-2.2), HEK293T (ATCC, CRL-11268), VA13 (ATCC, WI38–3374), Huh 7, Hep G2 (ATCC, HB-8065), M2 (ATCC, CRL-2500), MEFs (ATCC, CRL2991), BHK-21 (ATCC, CCL-10), WRD (kind gift from Dr. Yunning Sun), and MDCK (ATCC, CCL-34) cells were maintained in Dulbecco’s modified Eagle’s medium (DMEM) medium (Life Technologies, 11995-065) containing 10% fetal bovine serum at 37 °C, 5% CO_2_. BxPC-3 (ATCC, CRL-1687) pancreatic cancer cells were cultured in Gibco RPMI 1640 medium supplemented with 10% fetal bovine serum at 37 °C, 5% CO_2._ HeLa-S3 cells were suspension cultured in Minimum Essential Medium (MEM) Eagle Joklik, w/L-Gln w/o Ca^2+^ (Life science), containing 10% fetal bovine serum at 37 °C, 5% CO_2_.

### HeLa-S3 cell nuclear extract preparation

HeLa-S3 nuclear extract was prepared as previously described^53^. Briefly, suspension cultured 10 L HeLa-S3 cells were harvested when cell density reached to 5.0 × 10^5^ cells/ml by low speed centrifugation (1000 g, 10 min). Cells were resuspended and broken with dounce homogenizer (B pestle). Nuclei were spun down at 25,000 g, 4 °C, for 20 min. The nuclei pellet was extracted with KCl to a final concentration of 0.24 M. The nuclear extract was ultracentrifuged at 40000 rpm (Beckman), 4 °C for 30 min. The supernatant was dialyzed twice against Buffer D (HEPES-KOH 20 mM, KCl 100 mM, EDTA 0.2 mM, Glycerol 20%, DTT 1 mM, PMSF 0.5 mM, 0.22 μm filtered, pH 7.9) for 4 hours. The extract was snap-frozen in liquid N_2_ and stored at −80 °C.

### RNA isolation

Total RNA from HeLa-S3 was isolated with Trizol reagent (Ambion) according to the manufacturer’s instruction.

### hTRIR cDNA synthesis

hTRIR cDNA was synthesized with SuperScript™ III Reverse Transcriptase (Invitrogen) according to the manufacturer’s handbook.

### Plasmid construction

hTRIR (C19orf43, NCBI-GeneID: 79002) open reading frame, nt 1–528 was amplified and inserted into pET-33b(+) prokaryotic expression vector. hTRIRA, hTRIRB, hTRIRC, and hTRIRD were constructed by insertion of nt 163–528, nt 163–444, nt 1–330, nt 331–528, into pET-33b(+) vector, respectively. All the primers in plasmid construction were listed as follows: hTRIR (EcoRI-F: 5′-tccgaattcgatggctgcccgagggag-3′, SalI-R: 5′-acgcgtcgactcatttcaccaggggcc-3′), hTRIRA (EcoRI-F: 5′-tccgaattcgggcgtgaacttgttcgc-3′, SalI-R: 5′-acgcgtcgactcatttcaccaggggcc-3′), hTRIRB (EcoRI-F: 5′-tccgaattcgggcgtgaacttgttcgc-3′, XhoI-R: 5′-ccgctcgaggtcacctttacttgttaatac-3′), hTRIRC (EcoRI-F: 5′-tccgaattcgatggctgcccgagggag-3′, XhoI-R: 5′-ccgctcgaggcccggaccgcccttcctcttc-3′), hTRIRD (EcoRI-F:5′-tccgaattcgtccacacttagcttcgtg-3′,SalI-R:5′-ccgctcgagtttcaccaggggccgag-3′)

### Protein expression and purification

Protein expression and purification were performed as previously described^54^. Briefly, bacteria were harvested and lysed. The lysate was centrifuged at 14,000 rpm for 60 min, 4 °C. The supernatant was filtered and loaded onto 5 ml HisTrap HP column (GE Healthcare). The eluted fractions by 250 mM KCl were loaded onto a 5 ml HiTrap SP HP column (GE Healthcare). The 250 mM KCl eluted fractions were concentrated to approximate 1 ml and loaded on Superdex 75 gel filtration column (GE Healthcare). The peaked elution were concentrated, snap-frozen with liquid nitrogen and stored at −80 °C in 20 μl aliquots.

### hTRIR polyclonal antibody preparation

Purified hTRIR protein was applied to immunize rabbit according to previously described^55^. 1 mg hTRIR was dissolved in 1 ml PBS and intracutaneously injected into the rabbit on days 0, 15, 30 and 45. After 60 days immunization, whole blood was collected for polyclonal antibody preparation.

### Western Blot

Cells were seeded into 60 mm culture plate. After 48 hours, cells were harvested when cell confluence reached to 90%. Cell lysate were separated with 15% SDS-PAGE, transferred to nitrocellulose and probed with hTRIR polyclonal antibody. Luminescent signals were detected with SYSTEM GelDoc XR + IMAGELAB (Bio-Rad).

### α-^32^P-GTP labeled T7 transcription

α-^32^P-GTP (PerkinElmer) labeled RNA was transcribed with T7 polymerase (Thermo scientific) according to manufacturer’s instruction.

### RNA digestion assay

T7 transcribed RNA, total cellular RNA or RNA oligos were incubated with different amount of purified hTRIR in digestion buffer (25 mM NaCl, 0.5 mM Tris pH 7.5, 2% (v/v) glycerol) at 37 °C for 30 min or indicated time. RNAs were harvested and separated by 7 M urea polyacrylamide gel electrophoresis (PAGE) or 1.5% agarose (Biowest) gel. T7 Transcribed RNA signals were detected by Typhoon cyclone (PerkinElmer). Total cellular RNA signals were detected by SYSTEM GelDoc XR+ IMAGELAB (Bio-Rad). Small RNA was stained with stain-all (Sigma) and scanned with Epson scanner.

### DNA digestion assay

Total amount of 1 μg different types of DNA were incubated with 0.1 to 5 μg of purified hTRIR protein at 37 °C for 30 min. DNA was separated with 1% agarose (Biowest) gel and detected with SYSTEM GelDoc XR + IMAGELAB (Bio-Rad).

### Statistical analysis

Experiments were repeated at least three times. Quantification data were presented by mean and stand deviation. Student’s *t* test was applied to determine differences in DNA and RNA digestion assay. p < 0.05 was significant and marked with *.

## Additional Information

**How to cite this article**: Xie, J. *et al*. Identification of an RNase that preferentially cleaves A/G nucleotides. *Sci. Rep.* **7**, 45207; doi: 10.1038/srep45207 (2017).

**Publisher's note:** Springer Nature remains neutral with regard to jurisdictional claims in published maps and institutional affiliations.

## Supplementary Material

Supplementary Information
