# Supplementary material for: Identification of an RNase that preferentially cleaves A/G nucleotides
Source: Sci Rep. 2017 Mar 21;7:45207. doi: 10.1038/srep45207 (PMC5359670; doi:10.1038/srep45207)
Supplement: Supplementary Information [file srep45207-s1.pdf]

Identification of an RNase that preferentially cleaves A/G nucleotides

Jumin Xie[1, 2] Zhen Chen[1] Xueyan Zhang[1, 2] Honghe Chen[1] Wuxiang Guan [1*]

[1] Center for Emerging Infectious Diseases, Wuhan Institute of Virology, Chinese Academy of Sciences, Wuhan 430071, Hubei, China.

[2] University of Chinese Academy of Sciences, Beijing, 100049, China.

[*]Correspondence should be addressed to W.G. (guanwx@wh.iov.cn or guanwx2000@hotmail.com).

Supplementary table S1. 3 independent mass spectrum analysis of hTRIR.

	Accession	Description	ΣCoverage	Σ# Proteins	Σ# Unique Peptides	# AAs	MW [kDa]
1	Q9BQ61	Uncharacterized protein C19orf43 OS=Homo sapiens GN=C19orf43 PE=1 SV=1 - [CS043_HUMAN]	7.95	1	1	176	18.4
		Sequence	# PSMs	# Proteins	# Protein Groups	# Missed Cleavages	
		EAPGPAGGGGGGSR	2	1	1	0	
2	Q9BQ61	Uncharacterized protein C19orf43 OS=Homo sapiens GN=C19orf43 PE=1 SV=1 - [CS043_HUMAN]	22.73	1	3	176	18.4
		Sequence	# PSMs	# Proteins	# Protein Groups	# Missed Cleavages	
		QRQEEPPPGPQRPDQSAAAAGPGDPKR	3	1	1	2	
		QRQEEPPPGPQRPDQSAAAAGPGDPKR	3	1	1	2	
		KGGPGSTLSFVGK	2	1	1	1	
		GGPGSTLSFVGK	2	1	1	0	
3	Q9BQ61	Uncharacterized protein C19orf43 OS=Homo sapiens GN=C19orf43 PE=1 SV=1 - [CS043_HUMAN]	54.55	1	9	176	18.4

Supplementary figure S1.

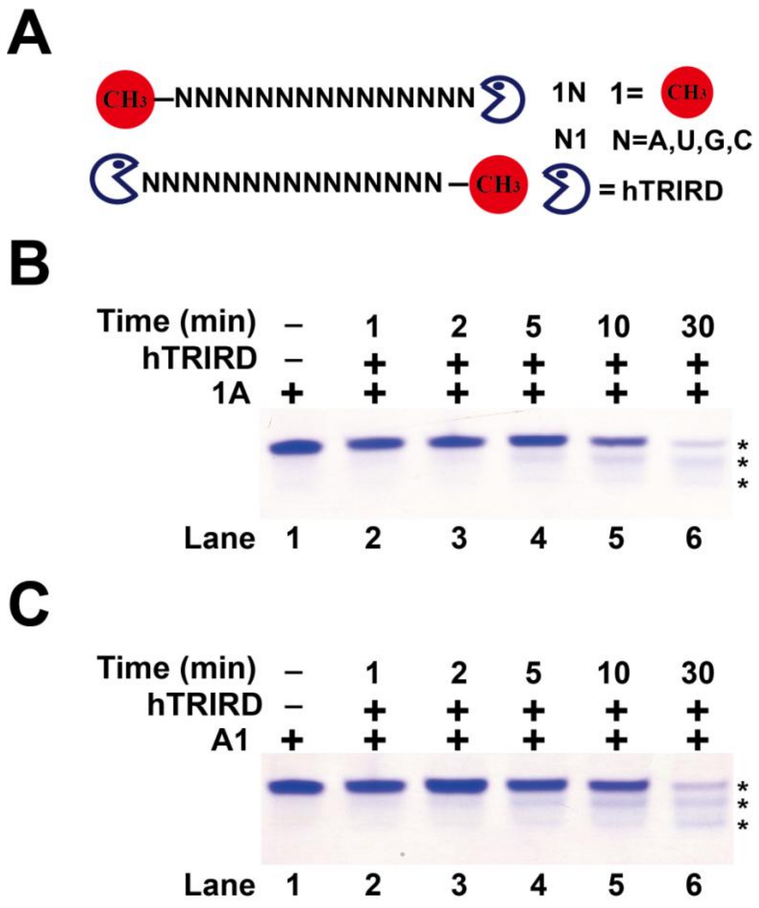

Figure S1. hTRIR is an exoribonuclease. (**A**) Chemically synthesized RNA oligos with methylated group at either 5’ end or 3’ end were

shown. (**B**) Synthesized 1A and (**C**) A1 were digested by hTRIRD for different time as indicated. Low molecular ladder of digested 1A and A1 were detected after 5 min incubation and marked with *.
